# Pre-diagnosis neutrophil-to-lymphocyte ratio and mortality in individuals who develop lung cancer

**DOI:** 10.1007/s10552-021-01469-3

**Published:** 2021-07-08

**Authors:** Laurie Grieshober, Stefan Graw, Matt J. Barnett, Gary E. Goodman, Chu Chen, Devin C. Koestler, Carmen J. Marsit, Jennifer A. Doherty

**Affiliations:** 1 Department of Population Health Sciences, Huntsman Cancer Institute, University of Utah, Salt Lake City, UT USA; 2 Department of Environmental Health, Rollins School of Public Health, Emory University, Atlanta, GA USA; 3 Program in Biostatistics, Division of Public Health Sciences, Fred Hutchinson Cancer Research Center, Seattle, WA USA; 4 Program in Epidemiology, Division of Public Health Sciences, Fred Hutchinson Cancer Research Center, Seattle, WA USA; 5 Department of Epidemiology, School of Public Health, University of Washington, Seattle, WA USA; 6 Department of Otolaryngology: Head and Neck Surgery, School of Medicine, University of Washington, Seattle, WA USA; 7 Department of Biostatistics & Data Science, University of Kansas Medical Center, Kansas City, KS USA; 8 Department of Population Health Sciences, Huntsman Cancer Institute, University of Utah, 2000 Circle of Hope Drive, Room 4746, Salt Lake City, UT 84112 USA

**Keywords:** Lung cancer, Non-small cell lung cancer, Small cell lung cancer, NLR, Methylation, Mortality

## Abstract

**Purpose:**

The neutrophil-to-lymphocyte ratio (NLR) is a marker of systemic inflammation that has been reported to be associated with survival after chronic disease diagnoses, including lung cancer. We hypothesized that the inflammatory profile reflected by pre-diagnosis NLR, rather than the well-studied pre-treatment NLR at diagnosis, may be associated with increased mortality after lung cancer is diagnosed in high-risk heavy smokers.

**Methods:**

We examined associations between pre-diagnosis methylation-derived NLR (mdNLR) and lung cancer-specific and all-cause mortality in 279 non-small lung cancer (NSCLC) and 81 small cell lung cancer (SCLC) cases from the β-Carotene and Retinol Efficacy Trial (CARET). Cox proportional hazards models were adjusted for age, sex, smoking status, pack years, and time between blood draw and diagnosis, and stratified by stage of disease. Models were run separately by histotype.

**Results:**

Among SCLC cases, those with pre-diagnosis mdNLR in the highest quartile had 2.5-fold increased mortality compared to those in the lowest quartile. For each unit increase in pre-diagnosis mdNLR, we observed 22–23% increased mortality (SCLC-specific hazard ratio [HR] = 1.23, 95% confidence interval [CI]: 1.02, 1.48; all-cause HR = 1.22, 95% CI 1.01, 1.46). SCLC associations were strongest for current smokers at blood draw (Interaction *P*s = 0.03). Increasing mdNLR was not associated with mortality among NSCLC overall, nor within adenocarcinoma (*N* = 148) or squamous cell carcinoma (*N* = 115) case groups.

**Conclusion:**

Our findings suggest that increased mdNLR, representing a systemic inflammatory profile on average 4.5 years before a SCLC diagnosis, may be associated with mortality in heavy smokers who go on to develop SCLC but not NSCLC.

**Supplementary Information:**

The online version contains supplementary material available at 10.1007/s10552-021-01469-3.

## Introduction

Lung cancer is the leading cause of cancer death worldwide [1], with five-year relative survival of 24% for non-small cell lung cancer (NSCLC), which primarily includes adenocarcinoma and squamous cell histotypes, and 6% for small cell lung cancer (SCLC) [2]. More than 65% of NSCLC and 90% of SCLC patients are diagnosed at an advanced stage [3], with five-year relative survival rates of just 6% for NSCLC and 3% for SCLC among those diagnosed at an advanced stage [2]. Patient factors before diagnosis that are associated with poorer lung cancer survival include older age, male sex, weight loss, and cigarette smoke exposure [4, 5].

It is well established that inflammatory processes are associated with risk of lung cancer [6–9], and it is plausible that an individual’s systemic inflammatory profile prior to a lung cancer diagnosis may be associated with mortality. In support of this hypothesis, inflammatory conditions such as chronic bronchitis and emphysema, chronic obstructive pulmonary disease (COPD), interstitial lung disease, and diabetes are associated with increased lung cancer mortality independent of their associations with increased lung cancer risk [10–19].

Elevated neutrophil-to-lymphocyte ratio (NLR) is a marker of systemic inflammation and immune stress that has been reported to be associated with all-cause mortality in most large prospective studies of healthy individuals [20–25], as well as cancer-specific mortality in one [20] of the three aforementioned studies in which cancer-specific mortality was also examined [20, 22, 25]. A single prospective study of healthy individuals observed that higher NLR was associated with future mortality from lung cancer [26]. In cancer patients with solid tumors, higher NLR (typically assessed prior to treatment) is an independent predictor of poor prognosis regardless of treatment strategy [27–30], and recent meta-analyses confirm that these associations hold in NSCLC and SCLC [31–37]. Higher NLR has been reported to be associated with greater smoking exposure in healthy populations that include never smokers, as well as with older age, male sex, and higher body mass index (BMI) [22, 38–41].

NLR can be easily quantified using results from simple hematology testing, specifically the complete blood count (CBC) with leukocyte differentials [42]. Though traditional CBC measurement cannot be performed on archival blood samples, lineage-specific DNA methylation patterns across the genome can be leveraged to estimate blood cell proportions that can be used to calculate methylation-derived NLR (mdNLR) [43, 44]. NLR measured at lung cancer diagnosis most likely reflects the disease state and possibly progression [45, 46]. However, NLR measured years prior to diagnosis provides a snapshot of the systemic inflammatory profile, which in addition to a person’s health state, developed immune system, and underlying genetics, may include evidence of exposure to environmental and behavioral risk factors [47, 48]. We have previously reported that pre-diagnosis mdNLR was associated with an increased risk of NSCLC (Odds Ratio [OR] per unit increase = 1.30, 95% CI 1.03, 1.63) but not SCLC (OR per unit increase = 1.06, 95% CI 0.77, 1.47) in a nested case–control study of heavy smokers after rigorous control for smoking history [49]. In the present study, we examined whether pre-diagnosis mdNLR was associated with mortality in heavy smokers who later developed lung cancer, as well as differences by lung cancer histotype.

## Methods

Our study included 360 individuals diagnosed with NSCLC or SCLC between 1994 and 2013 from the multicenter β-Carotene and Retinol Efficacy Trial (CARET) of heavy smokers at high risk for lung cancer [50]. We have previously published on mdNLR and lung cancer risk in a subset of these cases [49]. The present analysis additionally includes cases that were not able to be matched to controls for the risk analysis in [49], those with unknown pathology through 2005 who were later classified as a specific histotype, and additional cases ascertained during passive follow-up from 2005 to 2013 (Supplementary Table 1), resulting in 279 NSCLC and 81 SCLC cases. The NSCLC cases include 148 adenocarcinomas, 115 squamous cell carcinomas, and 16 cases with histotype NSCLC, NOS.

We assayed DNA methylation in the archival whole blood samples using the Illumina HumanMethylationEPIC BeadArray, followed by standard normalization and preprocessing procedures, as described previously [49]. We estimated proportions of six blood cell types (B cell, CD4T, CD8T, natural killer (NK), neutrophil, monocyte) for each case in our normalized methylation dataset using constrained projection of the EPIC IDOL-optimized cell mixture deconvolution matrix with the “projectCellType_CP” function from the FlowSorted.Blood.EPIC package in R [44]. This is in contrast to our prior work in which we used a deconvolution method based on CpGs that were identified using the 450 K CpG array data [49]. Cell type estimates, and therefore mdNLR, obtained from the two arrays are highly correlated in our study (mdNLR Spearman *r* = 0.99, *P* = 7.0E-301) and in the literature [44]. We opted to use the now available EPIC-optimized method for cell type estimation in this publication since 69% of the EPIC-optimized CpGs are unique to the EPIC array [44]. Continuous mdNLR was calculated as the ratio of predicted neutrophil and lymphocyte (sum of B cell, CD4T, CD8T, and NK) proportions, and we discretized mdNLR into quartiles based on the distribution from all 360 cases (Q1 0.39–1.424, Q2 1.425–1.898, Q3 1.899–2.462, Q4 2.463–16.90), representing increasing levels of systemic inflammation.

We evaluated associations between pre-diagnosis mdNLR and lung cancer-specific and all-cause mortality using multivariable-adjusted Cox proportional hazards models fit separately for NSCLC, adenocarcinoma, squamous cell carcinoma, and SCLC histotypes. We defined time to event as years from lung cancer diagnosis to death or December 31, 2013, whichever occurred first. Stage data were not available for cases ascertained between 2005 and 2013 due to passive follow-up procedures implemented after 2005, nor for those whose medical records could not be otherwise obtained. Therefore, our models included a strata variable to allow for differing baseline hazards by early (stage I/II), late (stage III/IV), or unknown stage. Models were a priori adjusted for variables assessed at the time the blood samples for methylation assays were drawn, based on biologic plausibility, including age, sex, smoking status, pack years, and time between blood draw and diagnosis. We assessed study covariates, such as body mass index (BMI), enrollment year, intervention arm, occupational asbestos exposure, race, years since quit smoking, and cigarettes smoked per day for potential confounding of mortality models based on a ≥ 10% change in continuous mdNLR hazard ratio estimates for each histotype in the a priori adjusted models. No additional covariates were included in our final models based on this threshold. We assessed all final models with continuous mdNLR to examine linear associations, as well as quartiled mdNLR coded using dummy variables with Q1 as the reference category to examine the possibility of non-linear associations. We calculated tests of log-linearity of hazard ratios across increasing quartiles of mdNLR (*P*-trend) using contrast coefficients and the corresponding dummy-coded quartile mdNLR model coefficients [51]. We did not observe departure from the Cox proportional hazards assumption for any variable in our main models (Table 2, NSCLC or SCLC) according to Schoenfeld residual testing [52].

In SCLC models, we explored effect modification by age (dichotomized at the mean in SCLC, 64.1 years), intervention arm, sex, smoking history (dichotomized at the mean in SCLC, 59.3 pack years), smoking status, and time between blood draw and diagnosis (dichotomized at the mean in SCLC, 4.5 years) by performing stratified analyses of the final, adjusted models. We evaluated statistical interaction between the dichotomous stratification variables and continuous mdNLR using product term *P*-values. Interaction models for intervention arm were also adjusted for the respective first level variable when assessing interactions since intervention arm was not included in the final, adjusted models. We performed a sensitivity analysis of our main models, overall and by histotype, excluding individuals diagnosed within two years of blood draw. Analytical modeling was performed in SAS 9.4 (Cary, NC). Statistical significance was defined using a nominal level of *P* < 0.05 in two-sided tests.

## Results

Participant characteristics at blood draw are summarized in Table 1. The histotype distribution for the 360 cases was: adenocarcinoma (*N* = 148), squamous cell carcinoma (*N* = 115), NSCLC, NOS (*N* = 16), and SCLC (*N* = 81). Whole blood was collected on average 4.7 (range 0.1 to 19.3) years prior to diagnosis for NSCLC cases and 4.5 (range 0.02 to 10.5) years prior to diagnosis for SCLC cases. Cases were on average 64 years old at blood draw, mostly white, and had mean smoking histories ranging from 57 to 62 pack years. Approximately 40% of adenocarcinoma and SCLC cases were female compared to 23% of squamous cell cases. More than half (56%) of NSCLC and 73% of SCLC cases were diagnosed at late stage (III/IV), though stage was missing for 13–23% of cases. Median time from diagnosis to death was shortest for SCLC (8.4 months), and longest for squamous cell carcinoma (12 months).

**Table 1:** Characteristics of lung cancer cases by histotype

	NSCLC			SCLC
	All NSCLC^a^	Adenocarcinoma	Squamous cell	
	(*N* = 279)	(*N* = 148)	(*N* = 115)	(*N* = 81)
Age at blood draw, years; mean (SD)	64.2 (5.5)	64.2 (5.6)	64.5 (5.6)	64.1 (5.9)
45 to < 55; *N* (%)	18 (6)	10 (7)	8 (7)	5 (6)
55 to < 60; *N* (%)	43 (15)	22 (15)	18 (16)	14 (17)
60 to < 65; *N* (%)	91 (33)	52 (35)	31 (27)	24 (30)
65 to < 70; *N* (%)	78 (28)	37 (25)	38 (33)	25 (31)
≥ 70; *N* (%)	49 (18)	27 (18)	20 (17)	13 (16)
Age at diagnosis, years; mean (SD)	69.0 (6.0)	68.6 (5.8)	69.1 (5.9)	68.6 (6.0)
BMI^b^; mean (SD)	27.3 (4.8)	27.5 (4.8)	27.1 (4.9)	28.0 (5.1)
Normal (≥ 18.5 and < 25); *N* (%)	88 (32)	46 (31)	37 (32)	21 (26)
Overweight (≥ 25 and < 30); *N* (%)	113 (41)	58 (39)	47 (41)	39 (48)
Obese (≥ 30); *N* (%)	75 (27)	43 (29)	29 (25)	20 (25)
Enrollment year; *N* (%)				
1985–1986	18 (6)	12 (8)	4 (3)	3 (4)
1987–1988	13 (5)	6 (4)	5 (4)	5 (6)
1989–1990	67 (24)	38 (26)	23 (20)	19 (23)
1991–1992	125 (45)	60 (41)	59 (51)	40 (49)
1993–1994	56 (20)	32 (22)	24 (21)	14 (17)
Race White; N (%)	266 (95)	143 (97)	107 (93)	78 (96)
Sex, female; *N* (%)	93 (33)	63 (43)	26 (23)	33 (41)
Current smoker at blood draw; *N* (%)	187 (67)	89 (60)	87 (76)	51 (63)
Pack years at blood draw; mean (SD)	59.0 (21.9)	57.3 (20.0)	62.0 (24.5)	59.3 (21.7)
Years since quit smoking at blood draw^c^; mean (SD)	6.6 (5.0)	6.2 (5.2)	7.3 (4.3)	6.0 (4.8)
Active intervention arm; *N* (%)	144 (52)	77 (52)	59 (51)	43 (53)
Asbestos exposure; *N* (%)	49 (18)	24 (16)	23 (20)	11 (14)
Stage; *N* (%)				
Early stage (I/II)	75 (27)	37 (25)	38 (33)	3 (4)
Late stage (III/IV)	156 (56)	85 (57)	62 (54)	59 (73)
Unknown	48 (17)	26 (18)	15 (13)	19 (23)
Months from diagnosis to death or end of follow-up^d^; median [IQR]	10.8 [39.6]	10.8 [45.6]	12.0 [39.6]	8.4 [9.6]
Years between blood draw and diagnosis; mean (SD)	4.7 (3.1)	4.5 (2.9)	4.6 (2.4)	4.5 (2.7)
mdNLR; mean (SD)	2.22 (1.52)	2.11 (1.22)	2.38 (1.91)	2.08 (1.32)

*BMI* body mass index, *IQR* interquartile range, *mdNLR* methylation-derived neutrophil-to-lymphocyte ratio, *NSCLC *non-small cell lung cancer, *NOS* not otherwise specified, *SCLC* small cell lung cancer, *SD* standard deviation

^a^"All NSCLC" includes adenocarcinoma, squamous cell, and 16 cases with histotype NSCLC, NOS

^b^BMI is missing for two participants (*N* = 1 NSCLC/squamous cell and *N* = 1 SCLC) and two NSCLC participants were underweight (*N* = 1 Adeno, *N* = 1 Squamous cell). BMI cut-points per National Heart, Lung, and Blood Institute definition: underweight (< 18.5), normal (≥ 18.5 and < 25), overweight (≥ 25 and < 30), and obese (≥ 30)

^c^Former smokers only

^d^Through December 31, 2013

Among SCLC cases, we observed a statistically significant 23% increased lung cancer-specific mortality (hazard ratio [HR] = 1.23, 95% confidence interval [CI]: 1.02, 1.48) and 22% increased all-cause mortality (HR = 1.22, 95% CI 1.01, 1.46) for each unit increase in pre-diagnosis mdNLR (Table 2). We observed similar results for quartiled mdNLR, with Q4 vs Q1 mdNLR HRs of 2.49 (95% CI 1.15, 5.40) for SCLC-specific mortality and 2.44 (95% CI 1.13, 5.26) for all-cause mortality. We observed a linear trend across increasing mdNLR quartiles for increased SCLC-specific and all-cause mortality (*P*-trends = 0.04). For all NSCLC cases and the adenocarcinoma and squamous cell carcinoma sub-histotypes, there were no patterns of association with continuous mdNLR and lung cancer-specific mortality (HR = 0.96, 95% CI 0.87, 1.05; HR = 1.02, 95% CI 0.86, 1.20; HR = 0.92, 95% CI 0.81, 1.04, respectively) or Q4 vs Q1 mdNLR (HR = 1.04, 95% CI 0.70, 1.54; HR = 1.20, 95% CI 0.71, 2.04; HR = 0.71, 95% CI 0.37, 1.34, respectively). Results were similar for all-cause mortality. Our sensitivity analysis restricting to individuals diagnosed two or more years after blood draw produced similar results (Supplementary Table 2). For SCLC, after excluding the 23% diagnosed within two years of blood draw, mortality estimates for Q4 vs Q1 were strengthened (SCLC-specific HR = 3.54, CI 1.37, 9.14; all-cause HR = 3.37, CI 1.33, 8.57), with similar estimates of linear trend (*P*-trends = 0.01 and 0.02, respectively); however, the continuous unit-change models were slightly attenuated (SCLC-specific HR = 1.21, CI 0.96, 1.53; all-cause HR = 1.19, CI 0.95, 1.50).

**Table 2:** mdNLR and mortality^a^ for lung cancer cases by histotype

mdNLR	NSCLC									SCLC		
	All NSCLC^b^			Adenocarcinoma			Squamous cell carcinoma					
	Death	Case	HR (95% CI)	Death	Case	HR (95% CI)	Death	Case	HR (95% CI)	Death	Case	HR (95% CI)
	*N*	*N*		*N*	*N*		*N*	*N*		*N*	*N*	
Lung cancer-specific mortality												
Continuous	224	279	0.96 (0.87, 1.05)	117	148	1.02 (0.86, 1.20)	94	115	0.92 (0.81, 1.04)	77	81	1.23 (1.02, 1.48)^*^
Q1 (lowest inflammation)	50	66	Ref	32	44	Ref	17	20	Ref	24	24	Ref
Q2	52	69	0.85 (0.57, 1.27)	24	32	0.85 (0.48, 1.52)	26	34	0.71 (0.36, 1.41)	20	21	1.60 (0.84, 3.02)
Q3	59	69	1.04 (0.70, 1.54)	28	32	1.06 (0.62, 1.81)	24	29	0.85 (0.42, 1.72)	18	21	1.41 (0.70, 2.82)
Q4 (highest inflammation)	63	75	1.04 (0.70, 1.54)	33	40	1.20 (0.71, 2.04)	27	32	0.71 (0.37, 1.34)	15	15	2.49 (1.15, 5.40)
	*P*-trend		0.63	*P*-trend		0.37	*P*-trend		0.41	*P*-trend		0.04
All-cause mortality												
Continuous	265	279	0.95 (0.88, 1.04)	137	148	0.97 (0.84, 1.12)	113	115	0.92 (0.83, 1.03)	80	81	1.22 (1.01, 1.46)^*^
Q1 (lowest inflammation)	61	66	Ref	39	44	Ref	20	20	Ref	24	24	Ref
Q2	65	69	0.86 (0.60, 1.24)	29	32	0.80 (0.48, 1.36)	34	34	0.77 (0.41, 1.44)	21	21	1.67 (0.89, 3.15)
Q3	67	69	1.03 (0.72, 1.48)	31	32	1.03 (0.63, 1.70)	28	29	0.87 (0.45, 1.66)	20	21	1.46 (0.74, 2.89)
Q4 (highest inflammation)	72	75	0.97 (0.67, 1.40)	38	40	1.10 (0.67, 1.81)	31	32	0.70 (0.38, 1.26)	15	15	2.44 (1.13, 5.26)
	*P*-trend		0.88	*P*-trend		0.50	*P*-trend		0.31	*P*-trend		0.04

*CI* Confidence Interval, *HR* Hazard Ratio, *mdNLR* methylation-derived neutrophil-to-lymphocyte ratio, *NSCLC* non-small cell lung cancer, *NOS* not otherwise specified, *SCLC* small cell lung cancer

^a^Mortality was estimated using Cox proportional hazards models adjusted for age, sex, smoking status, pack years at blood draw, and time between blood draw and diagnosis; stage (early (I/II), late (III/IV), unknown) was included as a strata variable

^b^"All NSCLC" includes adenocarcinoma, squamous cell, and 16 cases with histotype NSCLC, NOS

^*^Corresponding *P*-values = 0.02 for both continuous mdNLR models in SCLC cases

Stratified model results for continuous mdNLR and SCLC mortality are presented in Table 3. We observed stronger associations between mdNLR and SCLC-specific mortality in current smokers (HR = 2.00, 95% CI 1.32, 3.03) versus former smokers (HR = 1.14, 95% CI 0.83, 1.56), with interaction *P* = 0.03. We also observed stronger SCLC-specific mortality associations among those assigned to the placebo arm (HR = 1.86, 95% CI 1.29, 2.69) versus the active intervention (HR = 1.17, 95% CI 0.85, 1.60), and males (HR = 1.46, 95% CI 1.07, 1.98) versus females (HR = 1.09, 95% CI 0.85, 1.41), though these stratified results did not show evidence of statistical interaction (interaction *P*s ≥ 0.55). HRs for SCLC-specific mortality were similar in magnitude for strata defined by mean age at diagnosis, mean pack years, and mean time between blood draw and diagnosis. SCLC stratified all-cause mortality results were similar to those for SCLC-specific mortality.

**Table 3:** mdNLR and mortality^a^ for small cell lung cancer cases by subgroup

Variable	Strata definition	Lung cancer-specific mortality			All-cause mortality		
		Death	Case	HR (95% CI)	Death	Case	HR (95% CI)
		*N*	*N*		*N*	*N*	
Age at blood draw^b^	< 64.1 years	38	39	1.21 (0.96, 1.52)	38	39	1.21 (0.96, 1.52)
	≥ 64.1 years	39	42	1.22 (0.77, 1.95)	42	42	1.21 (0.77, 1.91)
	Interaction *P*^c^	0.88			0.98		
Intervention arm	Active	41	43	1.17 (0.85, 1.60)	42	43	1.17 (0.85, 1.60)
	Placebo	36	38	1.86 (1.29, 2.69)	38	38	1.74 (1.24, 2.42)
	Interaction *P*^c^	0.64			0.71		
Sex	Female	31	33	1.09 (0.85, 1.41)	33	33	1.10 (0.86, 1.42)
	Male	46	48	1.46 (1.07, 1.98)	47	48	1.49 (1.10, 2.02)
	Interaction *P*^c^	0.55			0.44		
Smoking history at blood draw^b^	< 59.3 pack years	41	44	1.12 (0.86, 1.46)	44	44	1.14 (0.88, 1.48)
	≥ 59.3 pack years	36	37	1.28 (0.97, 1.70)	36	37	1.28 (0.97, 1.70)
	Interaction *P*^c^	0.60			0.45		
Smoking status at blood draw	Former	28	30	1.14 (0.83, 1.56)	29	30	1.12 (0.83, 1.51)
	Current	49	51	2.00 (1.32, 3.03)	51	51	1.96 (1.30, 2.96)
	Interaction *P*^c^	0.03			0.03		
Time between blood draw and diagnosis^b^	< 4.5 years	39	41	1.35 (0.95, 1.91)	40	41	1.34 (0.95, 1.89)
	≥ 4.5 years	38	40	1.20 (0.93, 1.54)	40	40	1.16 (0.91, 1.49)
	Interaction *P*^c^	0.76			0.68		

*CI* Confidence Interval, *HR* Hazard Ratio, *mdNLR* methylation-derived neutrophil-to-lymphocyte ratio

^a^Mortality was estimated using Cox proportional hazards models adjusted for age, sex, smoking status, pack years at blood draw, and time between blood draw and diagnosis; stage (early (I/II), late (III/IV), unknown) was included as a strata variable

^b^Mean values among SCLC cases used to define strata

^c^Interaction *P*-value for the product term between continuous mdNLR and the dichotomized covariate; for intervention arm, both the main-effect and interaction terms were added to calculate the interaction *P* since intervention arm was not included in the final, fully adjusted models

## Discussion

To our knowledge, our study is the first to assess whether NLR estimated years before diagnosis is associated with mortality among individuals who go on to develop lung cancer. In this study of heavy smokers from CARET, we observed that pre-diagnosis mdNLR was associated with increased mortality for SCLC cases, but not for adenocarcinoma cases or squamous cell carcinoma cases.

Approximately 15% of lung cancer diagnoses are SCLC [2]. SCLC is the most aggressive lung cancer histotype with distinctive tumor behavior characterized by rapid growth, early and widespread metastases, genomic instability, and acquired chemoresistance [53]. Median survival in SCLC patients is just seven months [54]; we observed a median survival of 8.4 months in the 81 SCLC patients in our study. SCLC is not amenable to early detection by screening due to its short preclinical phase, so smoking cessation and improved treatments are the main targets for reducing mortality from this highly lethal and primarily smoking-related cancer [53, 55]. There are currently over 200 ongoing and recruiting clinical trials for SCLC, yet biomarkers for targeted therapy selection and immunotherapy in SCLC remain scarce [56].

NLR is an index of systemic inflammation that estimates the balance between the innate and adaptive immune systems [27]. Immune homeostasis is a complex and dynamic process that includes maintaining relatively constant component leukocyte proportions within physiologic ranges [47, 57]. Therefore, elevated NLR may indicate immune dysregulation that is evident from abnormal CBC components, such as high neutrophil or low lymphocyte counts, or the ratio measure may indicate low-grade immune dysregulation despite within-range CBCs. When measured at lung cancer diagnosis and prior to treatment, higher NLR is thought to reflect the disease state and likelihood of progression since higher neutrophil counts have been shown to promote metastasis [58–60], and lower lymphocyte counts have been observed to be associated with loss of tumor suppressor activities [61].

In our matched case–control study of heavy smokers from CARET [49], we observed that greater pre-diagnosis mdNLR was associated with increased risk NSCLC, but not SCLC. The present study, which evaluated whether mdNLR measured prior to diagnosis is associated with mortality among lung cancer cases, includes 240 NSCLC and 67 SCLC cases from our prior study [49]. Our present analyses also include one case from our prior study who was re-classified as NSCLC, NOS (from SCLC) after 2005, additional cases that were not able to be matched to controls in the prior study, and cases that accrued over additional follow-up time. There were no patterns of association between mdNLR and lung cancer-specific or all-cause mortality for NSCLC cases, nor among strata thereof. However, we did observe that higher pre-diagnosis mdNLR was associated with increased mortality for SCLC cases. Individuals in the highest quartile of mdNLR had 2.5-fold increased SCLC-specific mortality compared to those in the lowest quartile. Higher mdNLR was most strongly associated with increased SCLC-specific and all-cause mortality in current smokers, those assigned to the placebo arm, and males compared to each counterpart stratum.

The systemic inflammatory profile indicated by higher NLR could indicate a lesser ability to mount a robust immune response to a developing cancer and/or a favorable environment for the pathogenesis of more aggressive SCLC molecular histotypes [62, 63]. Given the short preclinical period of SCLC and the lack of association between mdNLR and SCLC risk in our previous work, we hypothesize that higher NLR measured years before a clinical SCLC diagnosis may reflect a systemic low-grade inflammatory profile that enables poorer post-diagnosis survival rather than occult carcinogenesis. Our sensitivity analysis excluding the 23% SCLC cases who were diagnosed within two years of blood draw supports this hypothesis since results were similar, and even stronger for comparisons of the top to the bottom mdNLR quartile, with a 3.5-fold increased SCLC-specific mortality in individuals diagnosed more than two years after their blood draw.

In the extensive literature on NLR and mortality in lung cancer patients, pre-treatment NLR is typically measured at diagnosis or up to 30 days prior to treatment [37, 64], and it has been reported to be associated with mortality in meta-analyses of both NSCLC and SCLC [31–37]. However, since blood was drawn on average 4.7 years (median 4.6 years) prior to lung cancer diagnosis in our study, these studies are not directly comparable to ours. One other study currently available in preprint is similar to our work in that respect—a study of 205 lung cancer cases from the “Give Us a Clue to Cancer and Heart Disease” cohorts (CLUE I/II), with mdNLR measured a median of 14 years prior to diagnosis [65]. They found that each standard deviation increase in pre-diagnosis mdNLR was associated with increased NSCLC-specific mortality (*N* = 149, HR = 1.50, 95% CI 1.19, 1.89). No results were presented for SCLC due to limited sample size (*N* = 29). In contrast to the CLUE I/II study, in which 10% of NSCLC cases were never smokers [65], our study only includes heavy smokers and our participants were older and had shorter times from blood draw to diagnosis. In addition, their mdNLR mean was lower, and standard deviations smaller, than those observed in the present study for NSCLC cases (CLUE I/II mdNLR mean 1.47 and SD 0.75; CARET mdNLR mean 2.22 and SD 1.52). Meta-analyses of pre-treatment NLR and mortality in lung cancer patients report NLR cut-offs for mortality associations between 2.2 and 5.9 [31–33], with a median NLR cut-off of 3.7 identified across 20 SCLC studies [34]. Thus, mdNLR in our study was more consistent with adult population-level estimates of NLR (from populations with respective mean ages 52 and 48 years) [38, 66]. We did not examine associations using the pre-treatment NLR literature-based cut-offs, as just 3.7% of the SCLC cases in our study had pre-diagnosis mdNLR > 5, and 4.9% had mdNLR > 3.7.

Though we were able to examine mortality within each histotype, specific histotype data were missing for 6% of NSCLC cases and stage data were missing for 19% of NSCLC or SCLC cases. Like most NLR studies, our study was limited by a single timepoint of estimated mdNLR. Given that NLR is dynamic in the presence of acute physiologic stress such as infections and disease development, any regression dilution bias in our prospective assessment would be expected to attenuate mortality associations similarly across histotypes [67]. So, while this bias may have impacted our ability to observe associations between mdNLR and mortality in NSCLC, adenocarcinoma, and squamous cell histotypes, the same bias would be expected to have likewise attenuated the magnitude of associations between mdNLR and SCLC mortality. We must be cautious in our interpretations of these findings, as we performed several statistical tests without adjusting the nominal *P*-value for multiple comparisons. Furthermore, our results have been obtained from a single observational study with a limited number of SCLC cases. Since CARET was a phase III chemoprevention trial, a major strength of our study was detailed participant and outcome data. Trial eligibility required that all participants have heavy smoking histories, making our study robust to confounding of the mdNLR and mortality associations by smoking.

Our results suggest that higher pre-diagnosis mdNLR, which may indicate a low-grade systemic inflammatory profile, is associated with poorer post-diagnosis survival following the most aggressive form of lung cancer, SCLC. Our study provides preliminary evidence suggesting that pre-diagnosis CBCs in heavy smokers at high risk of lung cancer could possibly be leveraged to provide patient-level information that ultimately may have applications in risk stratification as well as aiding clinical treatment choice and monitoring [45, 59].

## Supplementary Information

Below is the link to the electronic supplementary material.

Supplementary file1 (PDF 157 kb)

## Data Availability

The data that support the findings of this study are available from CARET but restrictions apply to the availability of these data, which were used in agreement with CARET for the current study, and so are not publicly available. Data are available from the authors upon request and with permission of CARET (http://www.compass.fhcrc.org/caretWeb/requests/requesting.aspx).
