# Supplementary material for: Pre-diagnosis neutrophil-to-lymphocyte ratio and mortality in individuals who develop lung cancer
Source: Cancer Causes Control. 2021 Jul 8;32(11):1227–36. doi: 10.1007/s10552-021-01469-3 (PMC8492578; doi:10.1007/s10552-021-01469-3)
Supplement: Supplementary file 1 — Supplementary file1 (PDF 157 kb) [file 10552_2021_1469_MOESM1_ESM.pdf]

Supplementary Tables

Supplementary Table 1. Summary of lung cancer cases (N=360) included in the present study by histotype.				
	**Adenocarcinoma**	**Squamous cell**	**NSCLC, NOS**	**SCLC**
Cases identified during active follow-up (1994 to 2005)				
Included in our risk analysis[a]	135	96	9	67[b]
Unable to be matched to controls for our risk analysis[a]	9	12	0	9
Cases with unknown histotype who were classified as a specific histotype post-2005	1	6	0	4
Case with SCLC histotype whose histotype was re-assigned post-2005	0	0	1[b]	0
Cases ascertained during passive follow-up (2005 to 2013)	3	1	6	1
**Total in the present study**	**148**	**115**	**16**	**81**

Abbreviations: NSCLC, NOS = Non-Small Cell Lung Cancer, Not Otherwise Specified; SCLC = Small Cell Lung Cancer

[a]Grieshober, L., et al. (2018). "Methylation-derived Neutrophil-to-Lymphocyte Ratio and Lung Cancer Risk in Heavy Smokers." Cancer Prevention Research **11**(11): 727-734.
[b]One SCLC case was re-classified as NSCLC, NOS for a total of 68 SCLC cases included in our risk analysis[a].

Supplementary Table 2. mdNLR and mortality[a] for lung cancer cases by histotype, excluding those diagnosed within two years of blood draw.

mdNLR	NSCLC									SCLC		
	All NSCLC[a]			Adenocarcinoma			Squamous cell carcinoma					
	Death N	Case N	HR (95% CI)	Death N	Case N	HR (95% CI)	Death N	Case N	HR (95% CI)	Death N	Case N	HR (95% CI)
**Lung cancer-specific mortality**												
**Continuous**	179	225	0.96 (0.87, 1.07)	86	112	1.10 (0.89, 1.36)	82	99	0.92 (0.81, 1.05)	59	62	1.21 (0.96, 1.53)
**Q1** (lowest inflammation)	44	58	Ref	27	38	Ref	16	18	Ref	20	20	Ref
**Q2**	37	51	0.80 (0.51, 1.25)	16	23	0.83 (0.43, 1.61)	20	26	0.69 (0.33, 1.46)	16	17	1.21 (0.58, 2.51)
**Q3**	46	54	1.07 (0.70, 1.63)	19	21	1.17 (0.63, 2.19)	21	26	0.92 (0.44, 1.92)	12	14	1.39 (0.60, 3.20)
**Q4** (highest inflammation)	52	62	0.98 (0.63, 1.51)	24	30	1.24 (0.67, 2.32)	25	29	0.66 (0.34, 1.30)	11	11	3.54 (1.37, 9.14)
	**P-trend**		0.75	**P-trend**		0.32	**P-trend**		0.38	**P-trend**		0.01
**All-cause mortality**												
**Continuous**	211	225	0.97 (0.89, 1.06)	101	112	1.12 (0.94, 1.35)	97	99	0.91 (0.81, 1.03)	62	62	1.19 (0.95, 1.50)
**Q1** (lowest inflammation)	53	58	Ref	33	38	Ref	18	18	Ref	20	20	Ref
**Q2**	47	51	0.86 (0.57, 1.29)	20	23	0.86 (0.47, 1.56)	26	26	0.80 (0.40, 1.60)	17	17	1.28 (0.63, 2.62)
**Q3**	52	54	1.07 (0.72, 1.59)	20	21	1.07 (0.60, 1.92)	25	26	0.99 (0.50, 1.99)	14	14	1.44 (0.64, 3.25)
**Q4** (highest inflammation)	59	62	0.94 (0.63, 1.41)	28	30	1.24 (0.70, 2.19)	28	29	0.65 (0.34, 1.24)	11	11	3.37 (1.33, 8.57)
	**P-trend**		0.95	**P-trend**		0.35	**P-trend**		0.29	**P-trend**		0.02

Abbreviations: BMI = Body Mass Index; CI = Confidence Interval; HR = Hazard Ratio; mdNLR = Methylation-Derived Neutrophil-to-Lymphocyte Ratio; NSCLC = Non-Small Cell Lung Cancer; NOS = Not Otherwise Specified; SCLC = Small Cell Lung Cancer

[a]Mortality was estimated using Cox proportional hazards models adjusted for age, sex, smoking status, pack years at blood draw, and time between blood draw and diagnosis; stage (early (I/II), late (III/IV), unknown) is included as a strata variable

[b]"All NSCLC" includes adenocarcinoma, squamous cell, and 16 cases with histotype NSCLC, NOS

Supplementary Table 3. Participating CARET Institutions and Federalwide Assurance Numbers by Study Center		
**Study center**	**Institution(s)**	**Federalwide Assurance Number (FWA)**
Seattle	Fred Hutchinson Cancer Research Center, Seattle, Washington	1920
	University of Washington, Seattle, Washington	6878
Baltimore	University of Maryland, Baltimore, Maryland	7145
Portland	Kaiser Foundation Research Institute on behalf of Kaiser Foundation Hospitals (Kaiser Permanente Center for Health Research, Portland, Oregon was the CARET-specific site)	2344
New Haven	Yale University, New Haven, Connecticut	2571
	Lawrence & Memorial Hospital, New London, Connecticut	3097
San Francisco	University of California, San Francisco, California	68
Irvine	University of California, Irvine, California	4071
